# Chemometric Assisted UV-Spectrophotometric Methods Using Multivariate Curve Resolution Alternating Least Squares and Partial Least Squares Regression for Determination of Beta-Antagonists in Formulated Products: Evaluation of the Ecological Impact

**DOI:** 10.3390/molecules28010328

**Published:** 2022-12-31

**Authors:** Ahmed Mostafa, Heba Shaaban

**Affiliations:** Department of Pharmaceutical Chemistry, College of Clinical Pharmacy, Imam Abdulrahman Bin Faisal University, King Faisal Road, P.O. Box 1982, Dammam 31441, Saudi Arabia

**Keywords:** multivariate curve resolution alternating least squares, PLS, beta antagonists, β-blockers, green analysis, UV-Vis spectrophotometry

## Abstract

In this study, UV-spectrophotometry coupled with chemometrics has been utilized to enhance the sustainability of quality control analysis of beta antagonists. First, we developed and optimized two eco-friendly chemometric-assisted methods without preliminary separation utilizing (1) multivariate curve resolution alternating least squares (MCR-ALS) and (2) well-established partial least squares regression (PLSR) multivariate calibration for the resolution and quantification of the most commonly prescribed beta antagonists in active pharmaceutical ingredients or commercial pharmaceutical products. The performance of the two proposed chemometric methods was computed and compared. Second, a comprehensive qualitative and quantitative evaluation of the eco-friendliness of the developed methods was performed utilizing the following greenness assessment tools: Green Analytical Procedure Index (GAPI), Analytical Eco-scale assessment (AES) tool, Raynie and Driver’s assessment tool and Analytical GREEnness Metric (AGREE). The models showed satisfactory recovery with a range from 99.83% to 101.12% for MCR-ALS and from 99.66% to 101.54% for PLSR. The optimized models were employed for green analysis of the investigated beta-blockers in single or co-formulated formulations without prior separation. The predictivity of the proposed MCR-ALS and the well-established PLSR method were very comparable. Nevertheless, the MCR-ALS method has the ability to recover the pure spectra of the studied analytes and the interferences as well. The proposed chemometric methods are fast, precise and do not need any sample pretreatment. In addition, they can be used as a benign substitute for the traditional methods used for the analysis of the investigated drugs in pharmaceutical products without harmful impacts on human health and the environment. They also provide advantages in terms of low solvent usage, reduced energy consumption and short analysis time, making them a safe and sustainable approach for quality control analysis.

## 1. Introduction

Analytical procedures are essential in various stages of drug manufacturing, including characterization of impurities, analysis of finished products or active ingredients, and determination of degradation products as well [1].

Hypertension is one of the most widespread diseases worldwide. Beta-antagonists (beta-blockers) are very effective in controlling blood pressure, hence minimizing the risk of cardiovascular diseases [2]. Bisoprolol (dihydrogen bis(1-[(propan-2-yl) amino]-3-(4-{[2-(propan-2-yloxy)ethoxy]methyl}phenoxy) propan-2-ol) (2E)-but-2-enedioate) (BS), atenolol (2-(4-{2-hydroxy-3-[(propan-2-yl) amino] propoxy}phenyl) acetamide (AT) and metoprolol (1-[4-(2-methoxyethyl) phenoxy]-3-[(propan-2-yl) amino]propan-2-ol) (MT) are cardio-selective beta-antagonists utilized for hypertension management and the control of myocardial infarction and cardiac arrhythmias [2]. Sotalol HCl (N-(4-{1-hydroxy-2-[(propan-2-yl) amino]ethyl}phenyl) methanesulfonamide hydrochloride) (ST) is a non-cardio-selective beta antagonist that is used for controlling both ventricular and supraventricular arrhythmias [2]. Hydrochlorothiazide (6-chloro-1,1-dioxo-3,4-dihydro-2H-1lambda6,2,4-benzothiadiazine-7-sulfonamide) (HZ) is a benzothiadiazine diuretic that blocks NaCl transport in distal convoluted tubule [2] and is widely used in combination with beta-blockers for treating high blood pressure. The therapeutic use of antihypertensive drugs, either individually or in combination, is increasing worldwide. Therefore, developing analytical methods capable of determining each of the active ingredients without the interference of the others is highly desired.

For routine analysis, chromatography is most commonly used. However, it consumes large volumes of organic solvents, produces large amounts of waste and requires sophisticated instruments [3]. On the other hand, spectrophotometric methods are simpler, cleaner, more eco-friendly and safer, which makes them green alternatives for chromatographic methods in most cases [3].

The lack of selectivity of classical spectrophotometric methods hinders their application in the presence of spectrum overlapping of different analytes [4]. The utilization of multivariate calibration methods allows for the high resolution of highly overlapped spectrums of multicomponent samples [5]. It is worth mentioning that partial least squares regression (PLSR) is one of the most widely used chemometric methods that could be effectively used to quantify target analytes because of its ability to overcome band overlapping and co-linearity [5]. The main advantage of partial least squares regression (PLSR) is the easiness of use; however, its performance highly relies on the selection methods of variables. For example, adding the most significant variables in the model will increase its potential to predict the desired properties in unknown samples [6].

Apart from the well-established multivariate calibration methods, e.g., PLSR, recently introduced multivariate curve resolution alternating least squares (MCR-ALS) methods are receiving much interest in the society of analytical chemistry. Multivariate curve resolution alternating least squares (MCR-ALS) offers advantages in terms of high resolution and the ability for the quantitative determination of analytes in complex matrices such as environmental samples [7], pharmaceuticals [5] and agricultural samples [8]. MCR-ALS provides high recovery of spectral information for both investigated analytes and other interferences [8].

Different methodologies have been introduced for the analysis of beta-antagonists either alone or with each other, e.g., [9,10,11,12,13]. However, the literature indicated that no analytical method had been previously reported for the concurrent determination of MT, AT, BI, ST and HZ. This promoted us to develop a simple, fast, cost-effective and eco-friendly method using widely available instruments (like a UV spectrophotometer) to determine these mentioned drugs simultaneously.

In this work, two chemometric-assisted methods employing MCR-ALS and PLSR chemometric models were proposed for the determination of the aforementioned drugs in their single and/or co-formulated products without prior separation steps. Additionally, the greenness profile of the developed methods was assessed using four assessment tools: (1) analytical eco-scale assessment (ESA) [14], (2) green analytical procedure index (GAPI) [15], (3) Raynie and Driver’s assessment tool [16] and (4) Analytical GREEnness Metric (AGREE) [17]. The proposed methods are green, economical, capable of analyzing a large number of samples in a short analysis time and amenable for routine analysis without harming the environment or causing hazardous effects on human health.

## 2. Experimental

### 2.1. Instrumentation

For spectrophotometric measurements, **a** UV-1800 Shimadzu double-beam spectrophotometer (Shimadzu, Kyoto, Japan) with a 1 cm quartz cell was used. The wavelength (from 200 to 400 nm) scanning speed was 2800 nm min^−1^. The bandwidth was set at 1 nm. Microsoft Excel was used for exporting spectra. PLS Toolbox software from Eigenvector Research, Inc., Manson, WA, USA (version 8.5) was employed for PLSR analysis. For MCR-ALS analysis, MCR-ALS GUI 2.0 software in combination with Matlab 2015a was utilized [18].

### 2.2. Material and Reagents

The standards of MT, AT, BS, ST and HZ were purchased from Sigma-Aldrich (Steinheim, Germany) with purity greater than 98%. Ultra-pure water system from ELGA, High Wycombe, UK, was employed for water purification. Methanol and HCl were purchased from Merck (Darmstadt, Germany). The band wavelength maxima for each studied analyte were as follows: 221.8 nm for MT, 224 nm for AT, 222 nm for BS, 227.4 nm for ST and 226 nm for HZ.

Commercial tablets include: Tenormin^®^ tablets from AstraZeneca, Egypt (50 mg of AT), Betacor^®^ tablets from Amoun Pharmaceutical Company, Egypt (80 mg of ST), Concor^®^ tablets from Amoun Pharmaceutical Company, Egypt (5 mg of BS), Betaloc^®^ tablets from AstraZeneca, Egypt (100 mg of MT) and Concor 5 plus^®^ tablets from Amoun Pharmaceutical Company, Egypt (5 mg of BS and 12.5 mg of HZ) were analyzed.

### 2.3. Preparation of Calibration and Standard Solutions

The stock solution of each investigated drug was prepared individually by accurately weighing ten mg of each analyte in methanol (10 mL) to get a stock solution of 1 mg mL^−1^. Most of the target analytes were not soluble in water except for metoprolol tartrate. Therefore, methanol was used for the preparation of the stock solutions. For the preparation of working solutions, water, methanol and 0.1N HCl were tested. In 0.1N HCl, the best results were obtained. Since β-blockers have high pKa, they exist in the ionized form in the acidic medium of 0.1N HCl. Therefore, 0.1N HCl was selected for further procedures. Working solutions were prepared by dilution from the stock solutions in 0.1 M HCl–water. The prepared solutions are stored at 4 °C. A set of 25 calibration solutions was formed using a multilevel multifactor design [18] covering the ranges of 4–14 µg mL^−1^ for MT, 2.5–10.5 µg mL^−1^ for AT, 0.5–4.5 µg mL^−1^ for BS, 1–7 µg mL^−1^ for ST and 0.5–5 µg mL^−1^ for HZ. The selected five-factors, five-levels design included orthogonal factors. In addition, a test set of nine samples containing the studied analytes (their concentration was randomly selected within the calibration range) was formed to be utilized as a validation set. Table 1 shows the calibration set and the validation set used for the studied drugs.

### 2.4. Sample Preparation

For each drug product, twenty tablets were individually weighed and finely ground. Then, a powder weight corresponding to the labeled weight of each drug tablet was added in 100 mL volumetric flasks and completely dissolved in methanol with the help of an ultrasonic bath for 10 min and then allowed to cool down at ambient temperature. The solution was filtered and diluted with 0.1 M hydrochloric acid.

## 3. Theoretical Background

### 3.1. MCR-ALS

In this section, a brief description of MCR-ALS will be provided, and for more detailed information, readers are referred to ref. [18,19]. Generally, MCR decomposes the data matrix to extract the most pertinent data of pure components in a mixture. This model is expressed as:

(1) $$D=CS^{T}+E$$

where D is the data matrix that contains the obtained spectra. C is the concentration matrix of the investigated analytes, and $S^{T}$ is the pure spectra matrix. E is the matrix of residuals (the data which is not identified by the model). The optimum component number can be determined using various methods, e.g., principal component analysis (PCA) [20].

The iterative process involves three steps: The first step is the decomposition of the D matrix (spectral data) into the bilinear matrix. The second step is the determination of the optimum number of components to ensure high resolution. The third step is the most crucial one, which involves the estimation of the concentration matrix and pure spectra matrix (C and $S^{T}$). In this study, the estimation was performed using simple-to-use interactive self-modeling mixture analysis (SIMPLISMA) [21].

The optimization of ALS depends on the utilization of various constraints, including non-negativity, closure and correlation constraints [22]. For the presented work, three constraints (non-negativity spectra, non-negativity concentration and correlation) were applied.

Non-negativity spectra constraint and non-negativity concentration constraint enforce the spectra and concentration profiles to be equal to or high than zero, respectively. Correlation constraint forces the building of an MCR-ALS model, which allows for the quantitation of the investigated analytes in the occurrence of other interferences [23].

Then, the proposed calibration models are employed for predicting the concentration in the validation set and the test set as well. After the predicted values are being updated, the iteration of ALS is finished, and the ALS optimization achieved when a specific convergence criterion is established [19]. The performance of the MCR-ALS model is evaluated using the % of lack of fit (Equation (2))

(2) $$lack\;of\;fit\;\left(\%\right)=100\sqrt{\frac{\sum_{i,j}e_{ij}^{2}}{\sum_{i,j}d_{ij}^{2}}}$$

where $d_{ij}$ is a component of the data matrix *D* and $e_{ij}$ is the residuals.

### 3.2. PLSR

PLSR decomposes the spectral (*D*) and concentration matrices (*c*) concomitantly according to the following equations:

(3) $$D=TP^{T\;}+E\;\;\;\;$$

(4) c=Tq+f           

where *T* is the matrix of scores, $P^{T}$ is the matrix loading, *q* is the vector loading, *E* is the residuals in D, and *f* is the residuals in *c*. The data was mean-centered, and the decomposition searches for latent vectors or factors, which greatly explained the covariance between D and c. Then, the decomposition of the spectral data matrix is employed for predicting the concentration matrix [24].

The selection of factors number in PLSR is a crucial step. For selecting the optimal number, the leave-one-out cross validation method was utilized in the presented study [25]. Twenty-five calibration samples were utilized for building the PLSR model. Calibration was carried out with 24 samples then the model was utilized to predict the sample which was left out. This process was replicated 25 times, and each sample was left out one time. The root mean square error of cross validation (RMSECV) was computed for each rotation.

### 3.3. Figures of Merit

For assessing the performance of the proposed MCR-ALS and PLSR models, validation samples were tested, and figures of merit such as Root mean square error of prediction (RMSEP), bias, standard error of prediction (SEP) and relative percentage error in the concentration predictions RE (%) were determined as per the equations:

(5) $$RMSEP=\sqrt{\frac{\sum_{i=1}^{n}{\left(c_{i}-{\hat{c}}_{i}\right)}^{2}}{n}}$$

(6) $$bias=\frac{\sum_{i=1}^{n}\left(c_{i}-{\hat{c}}_{i}\right)}{n}$$

(7) $$SEP=\sqrt{\frac{\sum_{i=1}^{n}{\left(c_{i}-{\hat{c}}_{i}-bias\right)}^{2}}{n-1}}$$

(8) $$RE\;\left(\%\right)=100\sqrt{\frac{\sum_{i=1}^{n}{\left(c_{i}-{\hat{c}}_{i}\right)}^{2}}{\sum_{i=1}^{n}c_{i}^{2}}}$$

where $c_{i}$ is the known concentration of analytes in sample i and ${\hat{c}}_{i}$ is the predicted concentration, n is the total number of samples forming the validation set. Additionally, a linear regression was performed between the known and predicted concentrations to calculate the correlation coefficient, slope and intercept.

## 4. Results and Discussion

**Figure 1:**
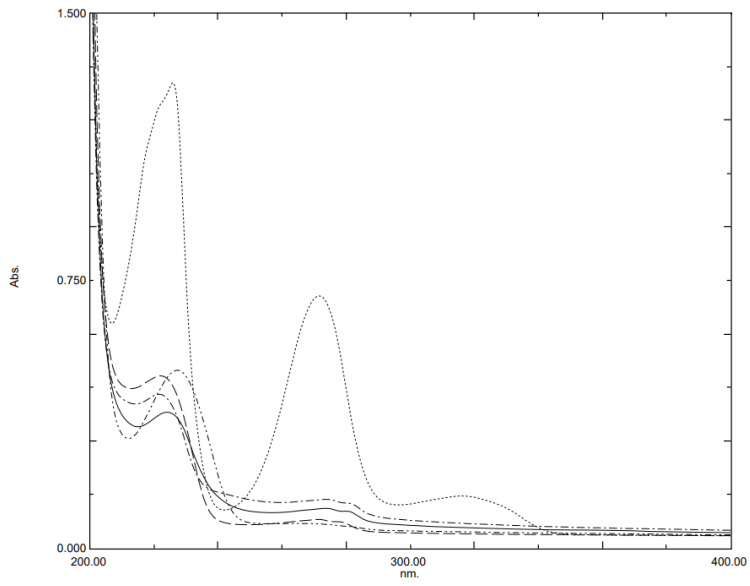
UV absorption spectra of 10 µg mL^−1^ of AT, ST, MT, BS and HZ.

Figure 1 presents the pure UV absorption spectra of the studied analytes MT, AT, BS, ST and HZ at the concentration of 10 µg mL^−1^. As illustrated in the figure, the overlapping of the spectra is highly noticeable; thus, their concurrent determination using simple procedures is unfeasible. Therefore MCR-ALS and PLSR chemometric methods were proposed for the determination of the studied drugs. MCR-ALS was first applied to synthetic experimental mixture samples for the resolution and quantification of the studied drugs.

### 4.1. Selection of Wavelength Intervals for MCR-ALS and PLSR

The selection of the optimum wavelength range is an essential step to ensure the quality of multivariate analysis. The absorbance data from the spectral region of 200–220 nm were eliminated due to the occurrence of noise, as shown in Figure 1. In addition, the region of 280–400 nm was excluded because the investigated beta-blockers have poor absorption in this region at the measured concentrations. The excipient (non-active) components in the tablets did not show any spectral absorbance in the wavelength range of 220 to 280 nm. Therefore, the wavelength range of 220–280 nm was found to be optimum for providing sufficient information on the investigated analytes; therefore, it was selected to quantify the studied drugs using MCR-ALS and PLSR models.

### 4.2. Multivariate Calibration

For constructing the calibration model, a multi-level multi-factor design [26] was applied. For the selected design, five levels were used for each factor which spans each other’s calibration space symmetrically and mutually orthogonal.

#### 4.2.1. MCR-ALS Model

**Figure 2:**
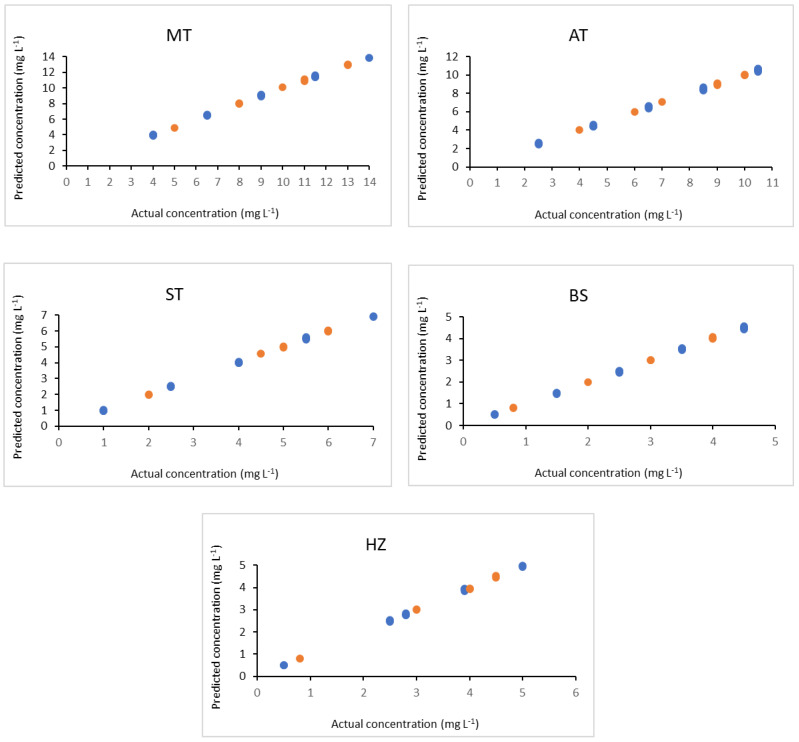
Plot of actual concentrations versus predicted values using MCR-ALS for calibration set (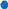) and validation set (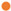) of the investigated drugs.

For preliminary estimation of pure analytes spectra in order to examine the resolution of MCR-ALS, a decomposition based on the SIMPLISMA algorithm was utilized, and five major components were selected [27]. MCR-ALS was applied to the calibration set (25 mixtures) for both spectra and concentration matrices using non-negativity constrain algorithm [27]. The maximum iterations number was maintained at fifty, but only eight iterations were needed to provide convergence. The variable which contains the quantitative information of the studied drugs was chosen and the correlation constraint was applied. The scatter plots of the actual concentrations versus the resolved concentration values determined by MCR-ALS are presented in Figure 2.

The high correlation coefficient (r^2^) values of ≥0.9990 and the low relative error (RE) of ≤1.49% were obtained for the investigated drugs, which proved that the predictive ability of the developed MCR-ALS model is high. The performance parameters of the proposed MCR-ALS model are illustrated in Table 2.

#### 4.2.2. PLSR Model

The calibration set used for MCR-ALS was also used for developing the well-established PLSR model. The data was mean-centered because of its higher sensitivity compared to the auto-scaling preprocessing method. Five latent variables were found to be optimum for the studied drugs, where the root mean square error of cross validation (RMSECV) was not significantly different from that of the next one. PLSR lacks the ability to accurately estimate the pure spectra of the studied analytes compared to MCR-ALS.

**Figure 3:**
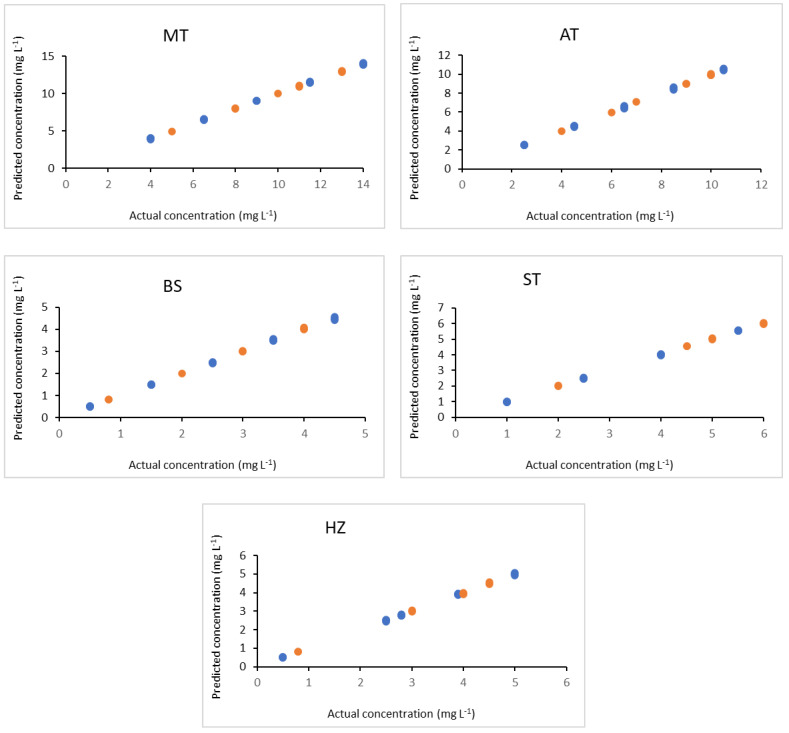
Plot of actual concentrations versus predicted values using PLSR for calibration set (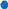) and validation set (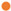) of the investigated drugs.

The developed PLSR model provided correlation coefficients ranging from 0.9993 to 0.9996, and low relative errors RE (%) ranged from 0.71% to 1.17%. The scatter plots of the actual concentrations versus the resolved concentration values determined by PLSR are presented in Figure 3. The figures of merit of the PLSR method are shown in Table 2. It was found that both developed models achieved good comparable results in terms of high correlation coefficients and low relative errors. The developed methods were then applied to predict the validation and test sets of different concentrations within the calibration range in order to evaluate the predictivity of the models.

### 4.3. Method Validation

Both developed MCR-ALS and PLSR models were employed for predicting the concentration of the studied drugs in 15 synthetic mixtures (forming an external validation set) with varying concentrations, as shown in Table 1. The constraints used for the calibration set were applied to the MCR-ALS algorithm. The PLSR data was mean-centered and employed for the validation set.

**Figure 4:**
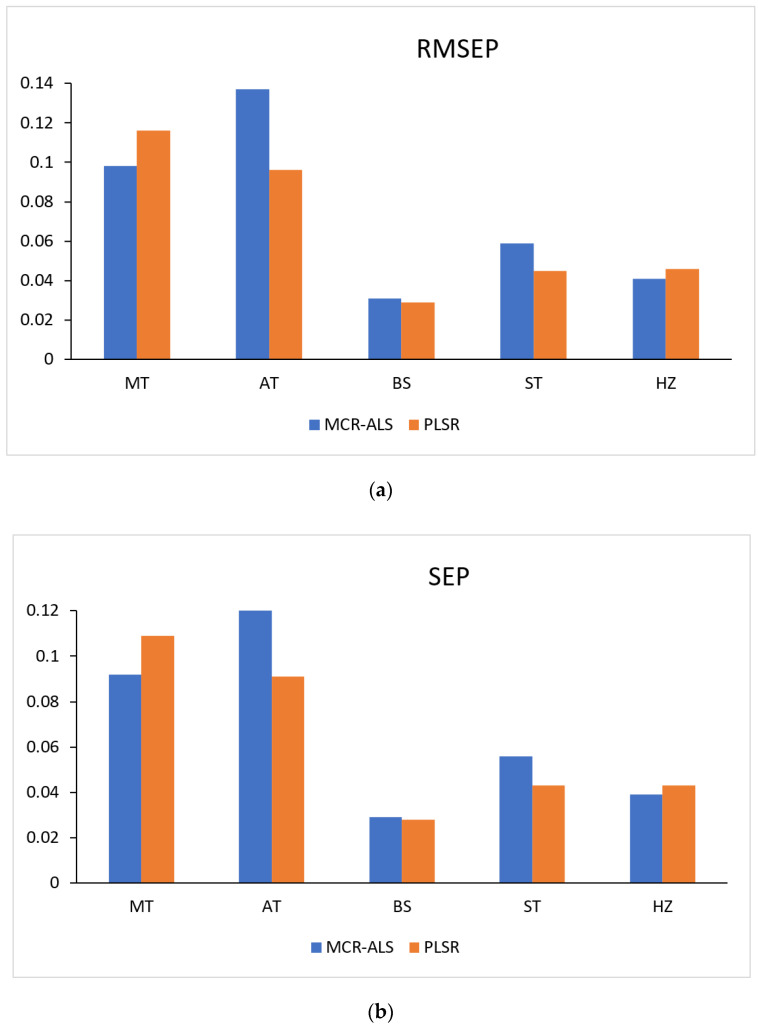
Bar charts for comparison of (**a**) RMSEP and (**b**) SEP values for the investigated drugs determined by applying the MCR-ALS and PLSR proposed methods.

Also, five latent variables were selected. The validation parameters, including RMSEP, r^2^, RE (%) and SEP, were determined to assess the performance of the MCR-ALS and PLSR models (Table 2). The comparison of the RMSEP and SD values determined by applying the proposed methods for the analysis of validation sets of the investigated drugs are presented in Figure 4. The figure indicated that the performance of the proposed methods was comparable. High correlation coefficient values ranging from 0.9990 to 0.9995 and from 0.9992 to 0.9996 were achieved for MCR-ALS and PLSR models, respectively. In addition, low relative errors RE (%) of ≤1.62% for MCR-ALS and ≤1.32% for the PLSR model were obtained. Furthermore, the linearity, range, precision, and accuracy were computed for both models, and the results are presented in Table 2. Satisfactory percent recoveries were achieved for MCR-ALS (≥ 99.84%) and the PLSR model (≥ 99.71%) as well.

The results showed that the predictivity of external validation for both methods was excellent, with no significant difference between the two developed models.

### 4.4. Analysis of Commercial Drug Products

To test the appropriateness of the presented chemometric models, they were tested for the determination of the investigated beta-blockers in drug products. Six replicate determinations were performed.

The obtained results are satisfactory and in good agreement with the label claims (Table 3). The results of the MCR-ALS model were statistically compared with that of the well-established PLSR model for concurrent determination of the studied drugs using Student’s t-test and F ratio at a 95% confidence level. It was shown that there was no significant difference between the two proposed methods (Table 3).

### 4.5. Assessment of the Environmental Impact of the Developed Methods

To ensure the safety of the developed methods on the environment and human health, the greenness profile of the methods was assessed. The utilization of two or more assessment tools was found to be an effective approach aimed at providing a comprehensive overview of the greenness level of analytical procedures [28]. Therefore, in this study, different evaluating tools such as the Analytical Eco-scale assessment (AES) tool, Raynie and Driver’s assessment tool, Green Analytical Procedure Index (GAPI) and Analytical GREEnness Metric (AGREE) were employed.

The AES tool is based on assigning penalty points to different items, including reagents (amount & hazard), instruments (energy & Occupational hazard) and waste (amount & treatment) [14]. After the summation of the penalty points, they are subtracted from 100 (base value) to give the final AES score of the analytical method. For the proposed methods, the AES score is 84, which indicated that the proposed methods were excellent green. The detailed calculation of penalty points and the final score of AES is illustrated in Table 4.

**Figure 5:**
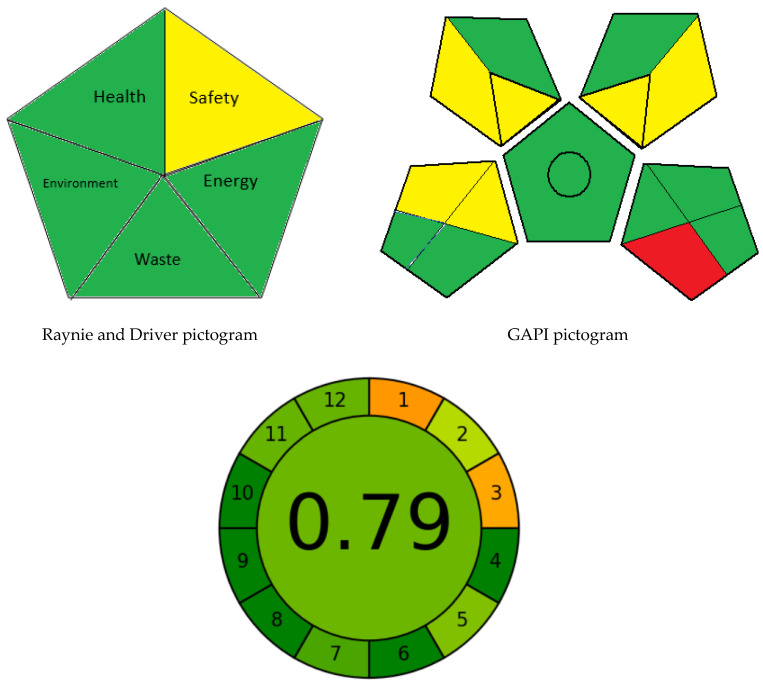
Assessment of the greenness profile of the developed methods using Raynie and Driver, GAPI and AGREE pictograms.

To complement the results given by AES, the pictogram developed by Raynie and Driver [16] was applied. This pictogram is divided into five parts representing energy, waste, environmental, safety and health. Each part is colored (green, yellow or red) according to the level of the method’s greenness. The parts which represent health and safety are based on the codes and standards of the National Fire Protection Association (NFPA) [29]. The environmental hazards and waste are measured according to the number of solvents used and produced during the analytical procedures. Measurement of energy depends on the method used (e.g., titration, HPLC, GC-MS, etc.). In this study, only one part was shaded yellow (pertaining to the safety: the NFPA flammability score of ethanol = 2), and four parts were colored green which indicated the greenness of the developed methods (Figure 5).

For quantitative and qualitative assessment of the greenness level of the proposed methods, Green Analytical Procedure Index (GAPI) [15] was employed. The GAPI pictogram consists of five pentagrams (15 sections) representing different parameters of analytical procedures covering the sample preparation, solvents, hazards of chemicals, instruments utilized, waste treatment and generation and energy consumed [15]. In this study, the GAPI pictogram contains eight green sections and only one red section (pertaining to waste treatment) (Figure 5). The overall GAPI profile of the presented methods confirmed their greenness and safety.

To complete the whole picture of the methods’ sustainability, the most recently introduced assessment tool, namely the Analytical Greenness Metric (AGREE), was employed. AGREE tool is the most reliable and informative evaluating tool, and it is highly recommended because of its simplicity and rapidity. It is an automated metric that can be used through user-friendly downloadable software [17]. This tool is presented as a circular pictogram composed of twelve parts representing the twelve principles of green analytical chemistry. Each part is shaded with a specific color from green to red depending on the level of the method’s greenness, and the final score of AGREE pictogram appears in the middle and ranges from 0 to 1, as illustrated in Figure 5. The final score of AGREE pictogram for the methods presented in this study is 0.79, which is very close to the maximum score of unity, hence indicating the greenness of the methods.

## 5. Conclusions

In this study, eco-friendly, fast and accurate chemometric methods were developed for concurrent spectrophotometric analysis of beta antagonists in single or co-formulated commercial formulations. The prediction ability of the proposed MCR-ALS and the well-established PLSR was comparable. Furthermore, MCR-ALS was able to recover the pure spectra of the investigated analytes and the interferences. The greenness profile of the proposed MCR-ALS and PLSR chemometric methods were investigated using Raynie and Driver’s, GAPI, Eco-scale and AGREE assessment tools. The proposed methods are found to be an environmentally friendly alternative to other traditional methods for the determination of beta-antagonists. It can also be employed for the quality control analysis of the studied drugs without prior preparation step.

## Figures and Tables

**Table 1:** The concentration matrix used for the calibration and validation sets of the studied drugs.

No.	Calibration Set (µg mL^−1^)					Validation Set (µg mL^−1^)				
	MT	AT	BS	ST	HZ	MT	AT	BS	ST	HZ
1	9.0	6.5	2.5	4.0	2.8	8.0	6.0	3.0	6.0	4.0
2	9.0	2.5	0.5	7.0	2.5	8.0	9.0	4.0	5.0	3.0
3	4.0	2.5	4.5	2.5	5.0	11.0	10.0	3.0	4.5	4.5
4	4.0	10.5	1.5	7.0	2.8	13.0	9.0	2.0	6.0	4.5
5	14.0	4.5	4.5	4.0	2.5	11.0	7.0	4.0	6.0	0.8
6	6.5	10.5	2.5	2.5	2.5	10.0	10.0	4.0	2.0	4.0
7	14.0	6.5	1.5	2.5	3.9	13.0	10.0	0.8	5.0	0.8
8	9.0	4.5	1.5	5.5	5.0	13.0	4.0	3.0	2.0	3.0
9	6.5	4.5	3.5	7.0	3.9	5.0	9.0	0.8	4.5	4.0
10	6.5	8.5	4.5	5.5	2.8					
11	11.5	10.5	3.5	4.0	5.0					
12	14.0	8.5	2.5	7.0	5.0					
13	11.5	6.5	4.5	7.0	0.5					
14	9.0	10.5	4.5	1.0	3.9					
15	14.0	10.5	0.5	5.5	0.5					
16	14.0	2.5	3.5	1.0	2.8					
17	4.0	8.5	0.5	4.0	3.9					
18	11.5	2.5	2.5	5.5	3.9					
19	4.0	6.5	3.5	5.5	2.5					
20	9.0	8.5	3.5	2.5	0.5					
21	11.5	8.5	1.5	1.0	2.5					
22	11.5	4.5	0.5	2.5	2.8					
23	6.5	2.5	1.5	4.0	0.5					
24	4.0	4.5	2.5	1.0	0.5					
25	6.5	6.5	0.5	1.0	5.0					

**Table 2:** Figures of merit of the developed MCR-ALS and PLSR models for the calibration and validation sets of the studied drugs.

	Calibration									
Parameters	MCR-ALS					PLSR				
	MT	AT	BS	ST	HZ	MT	AT	BS	ST	HZ
Calibration range (µg mL^−1^)	4.0–14.0	2.5–10.5	0.5–4.5	1.0–7.0	0.5–5.0	4.0–14.0	2.5–10.5	0.5–4.5	1.0–7.0	0.5–5.0
Intercept	−8.0 × 10^−3^	3.0 × 10^−12^	−8.0 × 10^−14^	−1.0 × 10^−13^	−5.0 × 10^−14^	3.4 × 10^−3^	4.6 × 10^−3^	1.4 × 10^−3^	1.9 × 10^−3^	1.4 × 10^−3^
Slope	1.0000	1.0000	1.0000	1.0000	1.0000	0.9996	0.9993	0.9994	0.9995	0.9995
Correlation coefficient (r^2^)	0.9994	0.9990	0.9994	0.9993	0.9994	0.9996	0.9993	0.9994	0.9995	0.9995
RMSECV	0.086	0.105	0.035	0.056	0.035	0.068	0.075	0.033	0.046	0.033
SEP	0.084	0.104	0.035	0.055	0.035	0.067	0.074	0.033	0.045	0.033
Bias	−4.17 × 10^−13^	7.59 × 10^−13^	8.72 × 10^−14^	−2.64 × 10^−13^	−2.62 × 10^−14^	1.2 × 10^−12^	4.00 × 10^−13^	−9.60 × 10^−13^	1.20 × 10^−13^	4.40 × 10^−13^
RE (%)	0.886	1.492	1.231	1.236	1.073	0.707	1.061	1.166	1.024	1.005
**Validation**										
**Parameters**	**MCR-ALS**					**PLSR**				
	**MT**	**AT**	**BS**	**ST**	**HZ**	**MT**	**AT**	**BS**	**ST**	**HZ**
Correlation coefficient (r^2^)	0.9990	0.9993	0.9995	0.9994	0.9994	0.9993	0.9994	0.9996	0.9992	0.9992
Accuracy (Mean ± SD)	99.84 ± 1.04	100.31 ± 0.70	100.54 ± 0.99	100.34 ± 0.93	99.89 ± 1.37	99.71 ± 0.94	99.93 ± 0.62	100.86 ± 1.15	100.47 ± 1.07	100.04 ± 1.55
Precision repeatability (RSD, %)	1.32	1.10	1.61	0.99	1.09	1.42	1.12	1.56	1.33	1.65
Intermediate precision (RSD, %)	1.42	1.25	1.54	1.11	1.21	1.56	1.20	1.35	1.05	1.50
RMSEP	0.098	0.137	0.031	0.059	0.041	0.116	0.096	0.029	0.045	0.046
SEP	0.092	0.129	0.029	0.056	0.039	0.109	0.091	0.028	0.043	0.043
Bias	−2.59 × 10^−3^	1.46 × 10^−3^	−0.013	−0.026	0.015	−0.012	−0.004	−0.017	−0.020	0.013
RE (%)	0.927	1.617	1.032	1.238	1.197	1.097	1.136	0.978	0.950	1.317

**Table 3:** Determination of the target analytes in commercial dosage forms using the proposed methods.

		MCR-ALS	PLSR
Sample 1	AT (Tenormin^®^ tablets)		
	Mean ± SD	101.06 ± 0.97	101.23 ± 0.78
	t	1.31	-
	F	1.51	-
	ST (Betacor^®^ tablets)		
Sample 2	Mean ± SD	101.02 ± 1.24	101.54 ± 0.71
	t	1.91	-
	F	2.25	-
	MT (Betaloc^®^ tablets)	100.46 ± 0.82	100.30 ± 0.71
Sample 3	Mean ± SD		
	t	1.34	-
	F	1.31	-
	BS (Concor^®^ tablets)		
Sample 4	Mean ± SD	100.09 ± 1.11	100.60 ± 0.90
	t	1.68	-
	F	1.53	-
Sample 5	BS (Concor 5 plus^®^ tablets)		
	Mean ± SD	101.10 ± 1.74	101.15 ± 1.01
	t	0.10	-
	F	2.95	-
	HZ (Concor 5 plus^®^ tablets)		
	Mean ± SD	99.72 ± 0.59	99.66 ± 0.75
	t	0.25	-
	F	2.16	-

SD: Standard deviation of the mean of the percentage recovery from the label claim amount. The theoretical values for t and F at (*p* = 0.05) are 2.13 and 6.39, respectively.

**Table 4:** The greenness profile of the developed MCR-ALS and PLSR chemometric methods employing Eco-scale tool.

1. Reagents			Penalty points (PPs)
1.1. Methanol	Amount	˂10 mL	1
	Hazard type	Single word: Danger	2
	Hazard amount	pictograms	3
			Total PPs = 6
1.2. Hydrochloric acid	Amount	˂10 mL	1
	Hazard type	Single word: Danger	2
	Hazard amount	2 pictograms	2
			Total PPs = 4
2. Instruments			
2.1. Energy (kWh/sample)	LC-UV	≤0.1	0
2.2. Occupational hazard		No hermetic sealing release of gas or vapor into air	0
3. Waste			
3.1. waste amount		1–10 mL	3
3.2. waste treatment		No treatment	3
Total penalty points			16
Eco-scale score			100−16 = 84

## Data Availability

Not available.

## Sample Availability

Not available.
